# Comparison of TST and IGRA in Diagnosis of Latent Tuberculosis Infection in a High TB-Burden Setting

**DOI:** 10.1371/journal.pone.0169539

**Published:** 2017-01-06

**Authors:** Surendra K. Sharma, Richa Vashishtha, L. S. Chauhan, V. Sreenivas, Divya Seth

**Affiliations:** 1 Department of Internal Medicine, All India Institute of Medical Sciences, New Delhi, India; 2 National Center for Disease Control (NCDC)^2^, New Delhi, India; 3 Department of Biostatistics, All India Institute of Medical Sciences, New Delhi, India; Indian Institute of Technology Delhi, INDIA

## Abstract

**Background:**

There are currently two tests for diagnosing latent tuberculosis infection (LTBI); TST and IGRA. However, it is still unclear that which one of these tests performs better in high TB-burden settings.

**Methods:**

1511 household contacts of pulmonary TB patients were enrolled to compare the performance of TST and IGRA for LTBI. At baseline all participant underwent testing for IGRA [QuantiFERON-TB® Gold In-tube (QFT-GIT) assay] and TST [2 tuberculin unit (TU), purified protein derivative (PPD), RT23, Staten Serum Institute (SSI), Copenhagen, Denmark]. All the household contacts were followed-up for two years for incident TB cases.

**Results:**

Active TB was diagnosed in 76 household contacts at an incidence rate of 2.14 per 1000 person-years. Both, TST [Hazard Ratio (HR): 1.14, 95% confidence interval (CI): 0.72–1.79, p = 0.57], as well as QFT-GIT assay (HR: 1.66, 95% CI: 0.97–2.84, p = 0.06) results at baseline were not significantly associated with subsequent development of active TB among household contacts of pulmonary TB patients.

**Conclusion:**

Neither TST nor IGRA predicted subsequent development of active TB among household contacts of pulmonary TB patients during follow-up. However, keeping in view the cost, and other logistics, TST remains the most preferred method for LTBI diagnosis in resource-limited, high TB-burden settings.

## Background

According to the World Health Organization (WHO) Global TB report, 2015 approximately one- third of the world population is infected with *Mycobacterium tuberculosis (Mtb*) [1]. Although individuals with LTBI are asymptomatic, however, they constitute an important reservoir contributing to the pool of active TB cases in future [2]. As the success of global TB control will heavily depend upon the performance of TB control programs of high TB-burden countries (HBCs), it is imperative to treat LTBI individuals along with active TB cases.

In view of the high background prevalence of LTBI, poor airborne infection control policies, development of drug resistance/toxicity and socioeconomic concerns, it has been emphasized that testing and treatment of LTBI in HBCs should be restricted to those who are at high-risk of progressing to TB disease[3]. Several published studies have informed that clustering of infectious TB cases within families increases the susceptibility of household contacts to LTBI and TB disease[4,5]. Therefore, identifications of household contacts of infectious TB cases can help curb progression and subsequent transmission,

The two currently available methods for diagnosis of LTBI include the century-old tuberculin skin test (TST) [5] and decade old immunodiagnostic test, interferon gamma release assays (IGRAs). Both these test work on the principle of cell mediated immunity[6]. It has been reported that the accuracy measures of TST are often confounded by Bacillus Calmette-Guérin (BCG) vaccination and non-tuberculous mycobacterial (NTM) infections. In an attempt to overcome to these limitations IGRAs utilizing region of difference-1 (RD-1) *Mtb* specific antigen were developed, which claimed to be more specific than TST[7]. However, due to absence of gold standard, there are limited data on diagnostic performance of these tests in LTBI.

Studies that compared the performance of these tests either using surrogate measures of sensitivity or specificity or index of exposure as the reference standard [8,9] are not clinically relevant and it remains unclear which test better identifies LTBI in HBCs. As LTBI testing intends to identify subjects who will eventually progress to develop active TB and would substantially benefit from preventive therapy, the accuracy of these tests can only be assessed by estimating their ability to predict active TB development. The present study was planned to compare the diagnostic performance of TST and QFT-GIT assay by following household contacts of pulmonary TB patients for two years with baseline QFT-GIT and TST done for a meaningful endpoint.

## Methods

### Study design and participants

This prospective and longitudinal study was carried out in the Department of Internal Medicine at the AIIMS, New Delhi, India which is a large tertiary care centre located in north India, with catchment area of several neighbouring states. The study was approved by the All India Institute of Medical Sciences, New Delhi ethics committee and written informed consent was obtained from all study participants. In case of children, written informed consent was obtained from their parents or legal guardians.

In this study, newly diagnosed HIV-negative, sputum-smear positive, pulmonary TB patients were recruited as index cases from the medical out-patient department (OPD) and Directly Observed Treatment Short-course (DOTS) centre of AIIMS, New Delhi and various other DOTS centre of Delhi region. Simultaneously, their close household contacts who were HIV-negative were enrolled in the study from January 2008 through March 2012. For the present study household contacts of pulmonary TB patients were defined as extended group of family members residing together with the pulmonary TB index case in the same household > 3 months and having a common cooking arrangement.

Subjects with past history of TB; HIV; hepatitis B and C positivity; pregnant and lactating women; existence of secondary immunodeficiency conditions such as diabetes mellitus, organ transplantation, malignancy and treatment with corticosteroids were excluded.

### Procedures

As per national TB guidelines the diagnosis of Category-I pulmonary TB index cases was confirmed through sputum-smear microscopy and *Mtb* culture on Löwenstein -Jensen (LJ) media [10]. Drug-susceptibility testing (DST) was also done to rule out drug-resistant TB cases.

Household contacts of pulmonary TB patients were screened for any symptoms and baseline investigations were done to rule out active TB. Contacts with cough (for more than 2 weeks), fever, night sweats, chest pain and weight loss were thoroughly examined by chest radiograph[11], sputum-smear for acid-fast bacillus (Ziehl–Neelsen staining method), sputum culture (LJ media by modified Petroff's method) and other investigations as indicated to rule out active pulmonary TB. In subjects with lymphadenopathy, organomegaly and serositis, appropriate imaging and relevant tests as deemed fit were done to rule out extrapulmonary TB (EPTB). Only subjects, without any signs and symptoms suggestive of active TB were recruited.

Data on basic demographic factors, tobacco smoking, alcohol consumption, family history of TB of all the study participants were captured in standardized pre-designed questionnaire (data in S1 File). Structured interview of index TB cases was carried out to ascertain about their household contacts status. Houses of pulmonary TB index patients were visited by trained field workers within two weeks of their enrolment to verify the physical condition of the household and to encourage eligible household contacts to participate in the study. Nutritional status was assessed by measuring body-mass index (BMI: kg/m^2^). The presence of Bacillus Calmette–Guérin (BCG) scar was noted. From smoking history of the study subjects smoking index or numbers of “pack-yr” were estimated [12,13] CAGE (cut-down, angry, guilty, eye-opener) questionnaire was administered for screening alcoholism among study subjects[14]. Data reflecting household contact exposure with the index patient (such as amount of time spent with the index patient during day time and night time, sleeping proximity with the index case etc.) and pertaining to the physical condition of the household (such as number of rooms in the house, number of door and windows/ventilation condition of the house etc.) were collected. The ventilation condition of the household was categorized as good, fair, or poor, based on information of the interviewer about the household conditions. They were referred as good, fair and poor when the average number of windows were six, three and one, respectively, and their average size were ten, six and four square meters, respectively.

At first visit, all participants underwent blood sampling for QFT-GIT assay (Cellestis, Ltd., Carnegie, Australia) and subsequently the tuberculin was administered intradermally into the volar surface of forearm. The TST was done using standardized 2TU, PPD, RT23 from SSI, Copenhagen, Denmark [courtesy Central TB Division (CTD), Ministry of Health and Family Welfare, Government of India] and the results were interpreted after 48 hours up to 72 hours by measuring the size of the induration (mm). The cut-off for TST was 10mm induration.

Study subjects were followed-up every six months for two years. These subjects were also provided with two telephone numbers and were asked to report back to hospital staff, at any time point of follow-up period, if any symptoms and signs of TB became apparent. Symptomatic subjects were intensively worked up for diagnosing active TB. They were thoroughly investigated clinically, microbiologically and with relevant imaging modalities to confirm TB breakdown as previously described^15-17^. They were classified as (i) *Definitive*- *Mtb* demonstrated in smear and/or culture or *Mtb*—PCR was positive in various body fluids (sputum, BAL, pleural fluid, ascitic fluid, pericardial fluid, CSF, bone marrow aspirates, pus specimens from cold abscesses) and (ii) *Probable*- specimen for smear and/or culture or *Mtb*-PCR was negative or cannot be obtained due to technical difficulties. The diagnosis of TB was made primarily on the basis of imaging or presence of exudative effusion or other body fluids with elevated adenosine deaminase activity (ADA) (>35 U/L).

### Laboratory assays

QFT-GIT format of IGRA was preferred over T-SPOT.*TB* assay (Oxford Immunotec Ltd., Oxford,United Kingdom) due to constrained resources and feasibility [as it is possible to store plasma samples and perform enzyme linked immunosorbent assay (ELISA) in batches].The QFT-GIT assay was performed as per the manufacturer’s instructions. For this assay three specialised blood collection tubes, namely: the nil control, TB antigen and mitogen tube available with test kit were utilized. The peripheral venous whole blood (3 ml) was collected through venepuncture from each participant directly into these three tubes of 1 ml each. These tubes were vigorously shaken to ensure through mixing of the blood with the content of the tubes. The TB antigen tube contained *M*.*tb* specific antigens ESAT-6, CFP-10 and TB7.7, used for stimulating the blood samples. The mitogen contained phytohemaglutinin (PHA) performed as a positive control. Tubes were transferred to the 37^0^ C incubator (within 6 hours of venepuncture) and incubated for 16–24 hours. Following incubation, plasma was harvested from the blood samples through centrifugation [2000–3000 relative centrifugal force (RCF)] for 15 minutes. The gel plugs present inside the tubes facilitated separation of the plasma from the blood cells. The plasma was collected and stored in properly labelled cryo-vials at -80^0^ till future use. The level of interferon-γ (IFN-γ) in plasms samples was estimated through enzyme linked immunosorbent assay [ELISA] kits (with recombinant human IFN-γ standard) provided with QFT-GIT assay packaging. The kit manufacturer’s protocol was followed. Proper thawing of the plasma samples were carried out, prior to ELISA which was performed manually in batches. Test result were interpreted as positive, negative or indeterminate as per kit manufacturer instruction.

### Statistical analysis

Stata version 12.0 (Stata Corporation, College Station, TX) was used for analysis. Two-sided *p*-value <0.05 was considered statistically significant. Characteristics of the study population were described using frequencies and percentages for categorical variables and the mean and standard deviation (SD) for quantitative variables. Risk factors for time to TB episode during follow-up (active TB development) were estimated using Cox proportional hazards regression.

## Results

A total of 1511 household contacts (age: 1–65 years) of 342 bacteriology confirmed pulmonary TB index patients (age: 18–65 years) were recruited in this study. Table 1 summarizes the baseline characteristics of enrolled household contacts.

**Table 1:** Baseline characteristics of the household contacts of pulmonary TB patients.

Variables	Household contacts (n = 1511)
**Age (years) [range]**	24.32 ± 15.18 [1–65]
**BMI (kg/m**^**2**^**)**	17.51 ±1.52
	n (%)
**Gender**	
Male	785 (52)
Female	726 (48)
**BCG scar**	
Present	1150 (76)
Absent	361 (24)
**Subjects coughing, duration (weeks)**	
≤ 2	71
3–5	210
≥ 6	16
**Tobacco-smoking**†	
Smokers	35 (2.3)
If yes, smoking index	
≤ 1 pack-yr	5 (0.3)
> 1 pack-yr	30 (02)
Non-smokers	1439 (95)
Status not known	37 (2.4)
**Alcoholic** ‡	
Yes	24 (02)
No	1377 (91)
Status not known	110 (07)
**TST**	
Positive	787 (52)
Negative	724 (48)
**QFT-GIT assay**	
Positive	917 (60)
Negative	581 (39)
Intermediate	13 (01)

Note: TB = tuberculosis; BMI = body mass index; BCG = Bacille Calmette-Guerin; DST = drug susceptibility testing; TST = tuberculin skin test; QFT-GIT = QuantiFERON-TB® Gold In-Tube.

Age and BMI is presented as mean + SD.

† In smokers smoking index or numbers of “pack-yr” were estimated as per definition provided by Malson et al. 2001. One pack-yr is defined as smoking of 20 cigarettes per day for one year. In India one pack of cigarette contains 10 cigarettes; therefore, smoking two packs of cigarettes per day for one year will be equivalent to one pack-year. Since, the net weight of tobacco in a bidi [150 to 240 mg] is about one-fourth of that in a cigarette, in bidi smokers; "cigarette equivalent pack years" were computed by dividing the "pack-yr" calculated on the basis of smoking bidis by four ^13^.

‡ Screening for alcoholism was done using CAGE criteria (C = Cut Down, A = Angry, G = Guilty, E = Eye opener), ^14^.

During two years follow-up of household contacts of pulmonary TB patients 76 [36 males (47%)] developed active TB. The mean ± SD age and BMI of these 76 TB cases were 22.83 ± 12.72 years and 16.08 ± 1.25 kg/m^2^ respectively. The median time to diagnosis of TB was 14.5 (11–15) months. Of 76 cases, 44 (58%) had definitive TB [41(54%) pulmonary TB and 03 (04%) extrapulmonary TB (EPTB)] and 32 (42%) had probable TB [09 (12%) pulmonary TB and 23(30%) EPTB] diagnosis as per definition provided earlier [15–17].

It can be seen from Table 2 that agreement between TST and BCG vaccination was higher among pediatric age group (between 1–14 years) as compared to subjects ≥ 15 years of age and there was a declining trend with increasing age. The overall agreement between TST results and BCG vaccination status in household contacts was higher [71.61%, kappa = 0.42 (95% CI: 0.38–0.46)] as compared to that obtained between QFT-GIT assay and BCG [60.95%, kappa = 0.11 (95% CI: 0.10–0.16)]

**Table 2:** Agreement of TST and QFT-GIT assay with BCG in household contacts of pulmonary TB.

Age Group (years)	BCG scar	TST		Agreement	Kappa (95%CI)	BCG scar	QFT-GIT		Agreement	Kappa (95%CI)
		Positive	Negative				Positive	Negative		
1–14 (n = 456)	Present	196	116	74.34%	0.51 (0.44–0.58)	Present	205	105	60.04%	0.12 (0.03–0.22)
	Absent	01	143			Absent	76	67		
15–24 (n = 435)	Present	202	140	65.98%	0.33 (0.27–0.43)	Present	224	117	63.74%	0.17 (0.08–0.26)
	Absent	08	85			Absent	40	52		
25–34 (n = 235)	Present	103	91	60%	0.25 (0.16–0.33)	Present	105	89	54.08%	0.05 (-0.05–0.15)
	Absent	03	38			Absent	18	21		
35–44 (n = 146)	Present	99	26	78.77%	0.39 (0.23–0.56)	Present	78	45	60.14%	0.02 (-0.12–0.16)
	Absent	05	16			Absent	12	08		
45–54 (n = 163)	Present	104	18	80.98%	0.52 (0.37–0.66)	Present	83	38	62.11%	0.10 (-0.06–2.53)
	Absent	13	28			Absent	23	17		
> 55 (n = 76)	Present	50	05	89.47%	0.74 (0.58–0.91)	Present	43	12	70.67%	0.27 (0.37–0.51)
	Absent	03	18			Absent	10	10		
**Total (n = 1511)**	Present	754	396	71.61%	0.42 (0.38–0.46)	Present	738	406	60.95%	0.11 (0.10–0.16)
	Absent	33	328			Absent	179	175		

Note. TB = tuberculosis; TST = tuberculin skin test; QFT-GIT = QuantiFERON-TB®Gold In-Tube; BCG = Bacille Calmette Guerin.

Table 3 estimates the incidence of active TB among 76 subjects with LTBI stratified according to age and TST and QFT-GIT assay results. The overall TB incidence rate was 2.14 per 1,000 person-years. The highest TB and lowest TB incidence rates were observed among contacts, who were QFT-GIT +ve TST–ve [3.70 (95% CI: 2.54–5.36)] and QFT-GIT-ve TST-ve [0.64 (95% CI: 0.29–1.42)] respectively. In TB incidence stratification based on age the highest TB incidence rate was observed among contacts between 25–34 years of age [2.91 (95% CI: 1.78–4.36)].

**Table 3:** Incidence of active TB among subjects with LTBI stratified by age and TST and QFT-GIT assay results (n = 1511).

Age group	TB cases N (%)	Incidence proportion	Person-years	Incidence rate [1000 person-years] (CI)
1–14 (n = 456)	20 (26.32)	4.39	10747.23	1.86 (1.20–2.88)
15–24 (n = 435)	27 (35.53)	6.21	10214.13	2.64 (1.81–3.85)
25–34 (n = 146)	16 (21.05)	6.81	5491.9	2.91 (1.78–4.76)
35–44 (n = 146)	06 (7.89)	4.11	3434.83	1.75 (0.78–3.89)
45–55 (n = 163)	04 (5.26)	2.45	3884.9	1.03 (0.39–2.74)
55–65 (n = 63)	03 (3.95)	3.95	1799.1	1.76 (0.57–5.46)
TST+ (n = 732)	42 (55)	5.74	18515.83	2.26 (1.68–3.07)
QFT-GIT+ (n = 917)	56 (74)	6.11	21515.27	2.60 (2.00–3.38)
TST- (n = 779)	34 (45)	4.36	17056.27	1.99 (1.42–2.79)
QFT-GIT- (n = 581)	19 (25)	3.27	13756.73	1.38 (0.88–2.16)
TST+QFT-GIT+ (n = 540)	29 (38)	5.37	14149.77	2.15 (1.42–2.94)
TST+ QFT-GIT- (n = 187)	13 (17)	6.95	4366.07	2.98 (1.75–5.13)
TST-QFT-GIT+ (n = 377)	28 (37)	7.42	7569.6	3.70 (2.55–5.36)
TST-QFT-GIT- (n = 394)	06 (08)	1.52	9390.67	0.64 (0.29–1.42)
Total (n = 1511)	76 (100)	5.03	35572.1	2.14 (1.71–2.67)

**Note.** TB = tuberculosis; CI = confidence interval; TST = tuberculin skin test; QFT-GIT = QuantiFERON-TB^®^Gold In-Tube.

Table 4 summarizes the hazard ratio for estimating various predictors of active TB development among household contacts of pulmonary TB patients. No significant association was observed between baseline TST and QFT-GIT assay results with development of active TB among household contacts. However, it was observed that malnutrition (BMI <18 kg/m^2^), tobacco smoking and poor household ventilation conditions were significantly associated with active TB development among the household contacts of pulmonary TB patients.

**Table 4:** Predictors of TB development among household contacts of pulmonary TB patients (n = 1511).

Participants’ characteristics	TB breakdown	No breakdown	Rate/1000 pyrs	HR(95%CI)	P	HR* (95%CI)	p
**Age group (yrs)**							
<35	63 (83)	1063 (74)	2.38	1			
≥35	13 (17)	372 (26)	1.42	0.60 (0.33–1.08)	0.09		
**Gender**							
Female	40 (53)	686 (46)	2.34	1			
Male	36 (47)	749 (52)	1.95	0.83 (0.53–1.30)	0.41		
**BMI (kg/m**^**2**^**)**							
≥ 18	07 (09)	761 (53)	0.38	1		1	
<18	69 (91)	674 (47)	4.01	10.62 (4.88–23.12)	<0.001	12.32(5.70–31.13)	<0.001
**BCG scar**							
Absent	13 (17)	348 (24)	1.52	1			
Present	63 (83)	1087(76)	2.33	1.54 (0.85–2.80)	0.15		
**TST**							
Negative	34 (45)	690 (48)	1.99	1			
Positive	42 (55)	745 (52)	2.27	1.14 (0.72–1.79)	0.57		
**QFT-GIT assay**							
Negative	19 (25)	562 (40)	1.38	1		1	
Positive	56 (75)	861 (60)	2.60	1.89 (1.22–3.18)	0.02	1.66 (0.97–2.84)	0.06
**Smoking**							
Absent	69 (93)	1370 (98)	2.03	1		1	
Present	05 (07)	30 (02)	6.42	3.24 (1.31–8.02)	0.01	6.17 (2.43–15.62)	<0.001
Ventilation condition*							
Good	03 (04)	229 (16)	0.54	1		1	
Average	26 (34)	759 (53)	1.40	2.60 (0.79–8.58)	0.12	2.16 (0.65–7.16)	0.21
Poor	47 (62)	447 (31)	4.11	7.66 (2.38–24.60)	0.001	6.36 (1.97–20.52)	<0.001
**Number of rooms in the house**							
>1	33 (43)	920 (64)	1.46	1	<0.001		
1	43 (57)	515 (36)	3.31	2.28 (1.45–3.59)	<0.001		
**Household members**							
<5	15 (20)	281 (20)	2.16	1			
5–8	48 (63)	950 (66)	2.04	0.94 (0.53–1.68)	0.85		
>8	13 (11)	204 (14)	2.55	1.18 (0.56–2.48)	0.66		
**HHCs & index case staying in**							
Different rooms	25 (33)	622 (43)	1.64	1			
Same rooms	51 (67)	813 (57)	2.51	0.08			
**HHCs night time exposure with index case (hrs)**							
**≤2**	19 (25)	619 (43)	1.26	1			
**>2**	57 (75)	816 (57)	2.79	2.37 (1.40–4.03)	<0.001		

**Note.** HHCs = household contacts; BMI = body mass index; BCG = Bacille Calmette Guerin; TST = tuberculin skin test; QFT-GIT = QuantiFERON-TB^®^ Gold In-Tube; HR = hazard ratio; CI = confidence interval; *Mtb* = *Mycobacterium tuberculosis*.

* HR obtained on multivariable analysis.

** Ventilation quality was categorized into good, fair, or poor, based on interviewers’ observation of household conditions such as the number and size of windows present.

†Radiographic disease severity was determined by National Diagnostic Standards and Classification of Tuberculosis. New York: National Tuberculosis Association, 1961.

‡Sputum-smear grading was done as per RNTCP guidelines 2010 ^12^.

## Discussion

In view of the WHO End TB strategy by 2035[1], it is important to deal with LTBI as it substantially adds to the TB disease burden. The recently published guidelines on management of LTBI are mainly intended for high-and upper-middle-income countries[18]. At present, treatment of active TB cases is the main priority for HBCs, however, eventually, these countries will have to deal with LTBI. The role of IGRAs and TST has not been systematically evaluated in low-income countries. The present study recruited a substantially large cohort of household contacts of pulmonary TB patients. These contacts were prospectively followed-up for two years for development of active TB after getting baseline QFT-GIT assay and TST done.

As reported earlier[19], there was higher QFT-GIT assay positivity [917 (60%)] as compared to TST [787 (52%)] among household contacts of pulmonary TB patients, however, it was not statistically significant. In the present study, both children [456 (30%)] and malnourished individuals [743 (49%)] were included and this could be a possible explanation for the diminished TST response owing to altered cellular- immunity in these subjects [20,21]. It was also observed that patients falling within the age group of 25–34 years had higher incidence rate as compared to pediatric patients. This could be attributed to the fact that subjects of this age group have exposure to *Mtb* in their house and also from the surroundings as they go out for work and other social activities.

Furthermore, in TB endemic settings, regular mucosal exposure to BCG or intake of several non-pathogenic environmental mycobacteria is also known to suppress TST response and increase mycobacteria-specific IFN-γ response in peripheral blood.[22]

Seventy six subjects among 1511 household contacts developed active TB. Exploration of TB incidence rate by TST and QFT-GIT assay result revealed higher TB incidence rate among QFT-GIT positive and TST negative [3.70 (95% CI: 2.55–5.36)] household contacts as compared to those TST positive and QFT-GIT negative [2.98 (95% CI: 1.73–5.13)]. Finding indicates that QFT-GIT assay may be marginally superior to TST in diagnosis of LTBI. A few recent studies also observed QFT-GIT performing marginally better than TST, however, they also advocate the prophylactic treatment being given to patients who are both QFT-GIT and TST positive. [23–25]

However, as the main goal of LTBI testing is to identify subjects who will eventually develop active TB, the performance of these tests was assessed by estimating their association with development of active TB through Cox proportional hazard regression analysis. Both estimates of the hazard ratios were although not statistically significant were comparable and pointed in the same direction. No significant association of baseline TST and QFT-GIT assay response with subsequent development of active TB in present study is consistent with previous systematic review showing poor predictive ability of both these tests.[26] The results suggest that TST and QFT-GIT assay, both being immunological markers of *Mtb* exposure, measuring cellular immune response to TB antigens have similar performance and may be used interchangeably. However, neither of them can accurately identify those infected and are imperfect for acting as an indicator for initiating preventive therapy among infected individuals.

In the present study, it was also observed that when either of these tests were positive, QFT-GIT/TST, the incidence of active TB was high as compared to when both these tests were negative. However, contacts who were both TST and QFT-GIT assay negative still developed active TB (although with lowest TB incident rate). These findings suggest reduced sensitivity and poor negative predictive value of these tests for progression to TB disease.

The declining trend of agreement between BCG vaccination and TST with increasing age in present study indicates waning effect of BCG vaccination with age. The limited effect of BCG vaccine after the age of 14, indicates that TST retains its specificity in HBCs.

Some inherent limitations of the present study include a small number of breakdown cases. A few more months of follow-up might have increased the number of breakdown cases and would have given a better understanding of the predicitive abilities of these tests. Furthermore, the high background prevalence of LTBI in HBCs does not allow us to differentiate between recent or remote infection.

In conclusion, neither of the tests predicted subsequent development of active TB in subjects with LTBI. However, currently as there are only two diagnostic tests available for diagnosis of LTBI, use of either test in HBCs can be recommended only after acknowledging their cost, logistics, population to be tested, and individual preference. In resource-limited and high TB burden countries, until we have a substantially improved test, TST should remain as the mainstay of LTBI testing due to low reagent cost, ease of applicability, no requirement of technical expertise, standardized labs and venepuncture. WHO in its first comprehensive guidelines on management of LTBI has also conditionally recommended TST for diagnosis of LTBI in low-and middle-income countries[18].

## Supporting Information

S1 File Questionnaire and Performa for subject recruitment. (DOC)
