# Supplementary material for: Comparison of TST and IGRA in Diagnosis of Latent Tuberculosis Infection in a High TB-Burden Setting
Source: PLoS One. 2017 Jan 6;12(1):e0169539. doi: 10.1371/journal.pone.0169539 (PMC5218498; doi:10.1371/journal.pone.0169539)
Supplement: S1 File — (DOC) [file pone.0169539.s001.doc]

**Questionnaire and Performa**

**Comparison of tuberculin skin test and interferon-γ release assay in latent tuberculosis infection and tuberculosis disease**

**Name**…………………………… ………………….

**Age**

**Sex** (0=F, 1=M)

**ID No**

**Date of Enrollment**

D D M M Y Y

**Parents / Guardian Name…**…………………………………………………………

**Address** ……………………………………………………………………………….

……………………………………………………………………………....................

**Telephone No: Land** …………………............ **Mobile** ………………………………..

**Occupation**……………………………………………………………………………….

**Education**…………………………………………………………………………………

**Married**

**Weight** **(kg)**

**Height (cm)**

**BMI(kg/m^2^)**

**HIV ELISA** (0=negative; 1= positive)

**WHO Clinical stage**  **(1/2/3/4)**

**Family History of TB**

**If yes,**

**Pattern /Type of TB** (1= PTB, 2=EPTB)

**BCG scar**

**Current PPD Skin Test induration, (mm)**

**Date injected**

D D M M Y Y

**Date read**

(after 48 hrs) D D M M Y Y

**Factors affecting the test**

- Corticosteroids
- cancer
- HIV infection
- Malnourishment

**(0=No, 1=Yes)**

**Interferon Gamma Release Assay:**

**Interpretation (1= positive, 2=indeterminate, 0=negative)**

| Date | Interpretation |
| --- | --- |
|  |  |
|  |  |
|  |  |
|  |  |
|  |  |

**Smoking**

(Cigarette **&** bidi)

If smoking yes,

Smoking index

(1 pack year =20 cigarettes/day/ 1year

In bidi smoker, 1 pack year estimated on

basis of smoking bidi /4

Exposure to second-hand smoke

***Alcohol**

*CAGE Questions: The patient should be asked four questions in the following manner. A positive response to any of the following questions will be considered an exclusion criterion.

1 Have you ever felt a need to CUT DOWN your drinking?

2 Have you ever felt ANGRY when confronted about the amount of alcohol you drink?

3 Have you ever felt GUILTY when confronted about the amount of alcohol you drink?

4 Have you ever felt the need to have a drink first thing in the morning? (EYE OPENER).

**Drug Abuse**

(0=None, 1=Injection -drug users, 2=inhalants)

**Risk Factors for Latent Tuberculosis Infection:**

- Persons with recent close contact with persons

known to have active tuberculosis

- Homeless person
- History of imprisonment
- History of exposure to TB patient at workplace

(especially) PTB

- Patients with known diabetes mellitus
- Biomass fuel user

**Symptoms: Duration in days**

Cough

Sputum production

Fever

Anorexia

Wt loss.

if yes, then quantify

**Patient group:**

**(1= HIV+LTBI+, 2=HIV+TB+, 3=HIV-TB+)**

If active TB yes**,**

Type of disease (1=PTB, 2=EPTB)

If EPTB site

(1=pleural effusion, 2= lymph node,

3=abdominal TB, 4= DTB 5=miliary TB,

6= meningeal TB 7= skeletal TB, 8= GUTB

and 9= pericardial effusion)

**(0=No, 1=Yes)**

If pleural effusion

(1=right, 2=Left, 3= bilateral)

**Method of Diagnosis**

(1= clinical only, 2= mycobacteriological only, 3= histopathological only, 4= radiological only, 5= molecular only)

**Radiological examination**

**Chest X-ray**

Date

D D M M Y Y

If abnormal, specify; Unilateral

Bilateral

Specify severity:

(1=Minimal lesions, 2=Moderately advanced, 3=Far advanced)

If advanced, specify: Cavitary

Non-Cavitary

Note:

Radiological classification of disease extent

1 Minimal: Minimal lesions include those that are of slight to moderate density but which do not contain demonstrable cavitations. They may involve a small part of one or both lungs, but the total extent , regardless of distribution , should not exceed the volume of lung on one side that occupies the space above the second chondrosternal junction and the spine of the forth or the body of the fifth thoracic vertebra.

2. Moderately advanced: Moderately advanced lesions may be present in one or both lungs, but the total extent should not exceed the following limits: disseminated lesions

of slight to moderate density that may extend throughout the total volume of one lung or the equivalent in both lungs; dense and confluent lesions limited in extent to one-third the volume of one lung; total diameter of cavitation, if present , must be less than 4cm.

3. Far advanced: Lesions more extensive than moderately advanced

**ULTRASOUND/ CT**

**Abdomen**

(If abnormal specify the findings)……………………………………………………….

………………………………………………………………………………………………

Hepatomegaly Focal lesion-liver

Splenomegaly Focal lesion-Spleen

Mesenteric LNs

RP lymph node

Ascites

Peripancreatic LNs

**CECT**

Chest

(If abnormal specify the findings)……………………………………………………….

………………………………………………………………………………………………

**Abdomen**)

(If abnormal specify the findings)……………………………………………………….

………………………………………………………………………………………………

**Head**

(If abnormal specify the findings)……………………………………………………….

………………………………………………………………………………………………

**Spine**

(If abnormal specify the findings)……………………………………………………….

………………………………………………………………………………………………

**(0=Normal, 1= Abnormal,2= Not done)**

**MRI**

**Head**

(If abnormal specify the findings)……………………………………………………….

………………………………………………………………………………………………

**Spine**

(If abnormal specify the findings)……………………………………………………….

………………………………………………………………………………………………

**Barium meal follow through**

(If abnormal specify the findings)……………………………………………………….

………………………………………………………………………………………………

**Mycobacteriological examination**

Sputum Smear for AFB

| Sample ID No | Dates | 3+ | 2+ | 1+ | Scanty bacilli | Negative |
| --- | --- | --- | --- | --- | --- | --- |
|  | Sample date  Result date |  |  |  |  |  |
|  | Sample date  Result date |  |  |  |  |  |
|  | Sample date  Result date |  |  |  |  |  |

[Definition

3 + = > 10 AFB/ oil immersion fields (no. of fields examined 20)

2 + = 1-10 AFB/ oil immersion fields (no. of fields examined 50)

1+ = 10-99 AFB in 100 oil immersion fields (no. of fields examined 100)

Scanty = 1-9 AFB in 100 oil immersion fields (no. of fields examined 200)

Negative = No AFB in 100 oil immersion fields (no. of fields examined 100)]

AFB culture (0=Negative, 1= Positive)

(If culture is positive then fill the following sensitivity test result)

Sensitivity (0=sensitive, 1=resistant)

Drug

Rifampicin

Isoniazid

Ethambutol

Pyrazinamide

**BAL/Pleural fluid/CSF**

If yes,

Smear for AFB(0=Negative, 1= Positive)

AFB culture (0=Negative, 1= Positive)

(If culture is positive then fill the following sensitivity test result)

Sensitivity (0=sensitive, 1=resistant)

Drug

Rifampicin

Isoniazid

Ethambutol

Pyrazinamide

ADA (U/L) (Pleural fluid/CSF)

**Histopathological examination**

**Tissue Diagnosis**

(0=FNAC, 1=Biopsy, 2=Not done)

Report

**(0=No, 1=Yes) ;(0=Normal, 1= Abnormal, 2= Not done)**

**Molecular examination**

**Sputum**

If yes

*M.tuberculosis* PCR (0=Negative, 1= Positive)

**BAL**

If yes

*M.tuberculosis* PCR (0=Negative, 1= Positive)

**Pleural fluid**

If yes

*M.tuberculosis* PCR (0=Negative, 1= Positive)

**CSF**

If yes

*M.tuberculosis* PCR (0=Negative, 1= Positive)

**Treatment**

**Anti TB Treatment**

If yes,

Date of Initiation

D D M M Y Y

TB –related outcome

(1=Cured, 2=Treatment completed,3=Defaulter, 4=Dead)

**ART**

If yes,

Date of Initiation

D D M M Y Y

Regimen

(1=AZT/3TC/ EFV Zidovudine/Lamivudine/Efavirenz

2=AZT/3TC/NVP Zidovudine/Lamivudine/Nevirapine

3=d4T/3TC/EFV Stavudine/Lamivudine/Efavirenz

4=d4T/3TC/NVP Stavudine/Lamivudine/Nevirapine)

Investigations

| **Date** (Baseline) |  |
| --- | --- |
| HB (gms/dl) |  |
| TLC(10^3^/Cumm) |  |
| DLC |  |
| Platelet count(10^3^/Cumm) |  |
| CD4 cell count |  |
| Viral load (HIV +ve) |  |
| ESR (mm/Ist Hour) |  |
| Glucose F (mg %) |  |
| PP (mg %) |  |
| R (mg %) |  |
| Urea (mg %) |  |
| Creatnine (mg %) |  |
| Uric acid (mg %) |  |
| Bil Total (mg %) |  |
| Conjugated (mg %) |  |
| Unconjugated (mg %) |  |
| Total Protein (g %) |  |
| Albumin (g%) |  |
| Globulin (g%) |  |
| SGOT(AST)I.U |  |
| SGPT(ALT)I.U |  |
| Alk. Phos(ALP) |  |

For Household contacts of family No

|  | **Contact 1** | **Contact 2** | **Contact 3** | **Contact 4 Contact 5** |
| --- | --- | --- | --- | --- |
| Name |  |  |  |  |
| Age |  |  |  |  |
| Sex (0= F, 1= M) |  |  |  |  |
| Contacts ID no. |  |  |  |  |
| Relation to patient |  |  |  |  |
| Date of Enrollment |  |  |  |  |
| Parents / Guardian Name |  |  |  |  |
| Telephone No |  | | | |
| Weight (Kg) |  |  |  |  |
| Height (cm) |  |  |  |  |
| BMI(kg/m^2^ |  |  |  |  |
| HIV ELISA (0=Negative, 1= Positive) |  |  |  |  |
| BCG scar |  |  |  |  |
| Current PPD skin test induration |  |  |  |  |
| Date injected |  |  |  |  |
| Date read |  |  |  |  |
| Factors affecting the test   - Corticosteroids - Cancer - Malnourishment |  |  |  |  |
| Interferon Gamma Release Assay:  (0=negative,1=positive,2=indeterminate)  Date | Reading | Reading | Reading | Reading Reading |
| Ventilation condition of the house  No. of windows in the house  Size of the windows  Height of the ceiling from the floor  No. of rooms in the house  No. of doors in the house  No. of family members staying in the house  No. of members staying in one room  Sharing room with the index case  Exposure to the index case in day time (in hrs)  Exposure to the index case in night time (in hrs)  Sleeping site relative to the index case Whether contact taking any preventive therapy  If yes,  Duration for which therapy is taken |  | | | |
| S Smoking  (Cigarette and bidi)  If smoking yes,  Smoking index  (1 pack year =20 cigarettes/day/ 1year  In bidi smoker, 1 pack year estimated on basis of smoking bidi/4)  Exposure to second-hand smoke |  |  |  |  |
| Alcohol (CAGE criteria) |  |  |  |  |
| Drug abuse (0=None, 1=Injection -drug users, 2=inhalants) |  |  |  |  |
| **Symptoms:**  Cough  Sputum production  Fever  Anorexia  Wt loss.  if yes, then quantify |  |  |  |  |
| **If symptoms suggestive of active TB,**  **Radiological examination**  **Chest X-ray** (0=Normal, 1= Abnormal**)**  If abnormal specify  Unilateral  Bilateral  Specify severity:  (1=Minimal lesions, 2=Moderately advanced, 3=Far advanced)  If advanced, specify:  Cavitary  Non-cavitary  Note:  Radiological classification of disease extent  1 Minimal: Minimal lesions include those that are of slight to moderate density but which do not contain demonstrable cavitations. They may involve a small part of one or both lungs, but the total extent , regardless of distribution , should not exceed the volume of lung on one side that occupies the space above the second chondrosternal junction and the spine of the forth or the body of the fifth thoracic vertebra.  2. Moderately advanced: Moderately advanced lesions may be present in one or both lungs, but the total extent should not exceed the following limits: disseminated lesions  of slight to moderate density that may extend throughout the total volume of one lung or the equivalent in both lungs; dense and confluent lesions limited in extent to one-third the volume of one lung; total diameter of cavitation, if present , must be less than 4cm.  3. Far advanced: Lesions more extensive than moderately advanced  **ULTRASOUND/ CT**  **Abdomen**  (If abnormal specify the findings)  **CECT**  **Chest**  (If abnormal specify the findings)  **Abdomen**  (If abnormal specify the findings)  **Head**  (If abnormal specify the findings)  **Spine**  (If abnormal specify the findings)  **MRI**  **Head**  (If abnormal specify the findings)  **Spine**  (If abnormal specify the findings)  **Barium meal follow through**  (If abnormal specify the findings) |  |  |  |  |
| **Mycobacteriological examination**  **(3+,2+, 1+ Scanty and Negative)**  **Sputum Smear for AFB**  Sample ID No  [Definition  3 + = > 10 AFB/ oil immersion fields (no. of fields examined 20)  2 + = 1-10 AFB/ oil immersion fields (no. of fields examined 50)  1+ = 10-99 AFB in 100 oil immersion fields (no. of fields examined 100)  Scanty = 1-9 AFB in 100 oil immersion fields (no. of fields examined 200)  Negative = No AFB in 100 oil immersion fields (no. of fields examined 100)]  AFB culture (0=Negative, 1= Positive)  (If culture is positive then fill the following sensitivity test result)  (0=sensitive, 1=resistant)  Drug  Rifampicin  Isoniazid  Ethambutol  Pyrazinamide  **BAL/Pleural fluid/CSF**  Smear for AFB(0=Negative, 1= Positive)  AFB culture (0=Negative, 1= Positive)  (If culture is positive then fill the following sensitivity test result)  (0=sensitive, 1=resistant)  Drug  Rifampicin  Isoniazid  Ethambutol  Pyrazinamide |  |  |  |  |
| **Histopathological examination**  **Tissue Diagnosis**  (0=FNAC, 1=Biopsy, 2=Not done)  Report |  |  |  |  |
| **Molecular examination**  **Sputum**  If yes  *M.tuberculosis* PCR (0=Negative, 1= Positive)  **BAL**  If yes  *M.tuberculosis* PCR (0=Negative, 1= Positive)  **Pleural fluid**  If yes  *M.tuberculosis* PCR (0=Negative, 1= Positive)  **CSF**  If yes  *M.tuberculosis* PCR (0=Negative, 1= Positive) |  |  |  |  |
| **Treatment**  **Anti TB Treatment**  If yes,  Date of Initiation |  |  |  |  |
| **Clinical monitoring**  Date  Cough  Expectoration  Weight loss  Appetite  Fever  node development  Sputum Smear  Sputum Culture  CXR  Imaging  ATT-Adherence(if on ATT) |  |  |  |  |
| **Investigations**  Date  HB (gms/dl)  TLC(10^3^/Cumm)  DLC  Platelet count(10^3^/Cumm)  CD4 cell count  Viral load (HIV +ve)  ESR(mm/Ist Hour)  Glucose F (mg %)  PP (mg %)  R (mg %)  Urea (mg %)  Creatnine(mg%)  Uric acid (mg %)  Bil. Total (mg %)  Conjugated (mg%)  Unconjugated(mg%)  Total Protein (g %)  Albumin (g %)  Globulin(g%)  SGOT(AST)I.U  SGPT(ALT)I.U  Alk. Phos(ALP |  |  |  |  |
